# Electrically driven three-dimensional solitary waves as director bullets in nematic liquid crystals

**DOI:** 10.1038/s41467-018-05101-y

**Published:** 2018-07-25

**Authors:** Bing-Xiang Li, Volodymyr Borshch, Rui-Lin Xiao, Sathyanarayana Paladugu, Taras Turiv, Sergij V. Shiyanovskii, Oleg D. Lavrentovich

**Affiliations:** 1 Liquid Crystal Institute and Chemical Physics Interdisciplinary Program, Kent State University, Kent, OH 44242 USA; 2 Department of Physics, Kent State University, Kent, OH 44242 USA

## Abstract

Electric field-induced collective reorientation of nematic molecules is of importance for fundamental science and practical applications. This reorientation is either homogeneous over the area of electrodes, as in displays, or periodically modulated, as in electroconvection. The question is whether spatially localized three-dimensional solitary waves of molecular reorientation could be created. Here we demonstrate that the electric field can produce particle-like propagating solitary waves representing self-trapped “bullets” of oscillating molecular director. These director bullets lack fore-aft symmetry and move with very high speed perpendicularly to the electric field and to the initial alignment direction. The bullets are true solitons that preserve spatially confined shapes and survive collisions. The solitons are topologically equivalent to the uniform state and have no static analogs, thus exhibiting a particle–wave duality. Their shape, speed, and interactions depend strongly on the material parameters, which opens the door for a broad range of future studies.

## Abstract (web summary)

Solitary waves which maintain their shape have many fascinating physical and mathematical properties. Here the authors observe waves trapped along three spatial dimensions in nematic liquid crystals due to an electric field and show that these director bullets can re-form after collisions.

## Introduction

Solitary waves are localized waves which propagate at a constant speed without changing their shape, thanks to a balance of dispersive and nonlinear effects. Some solitary waves can preserve their identities after pair-wise collisions, thus behaving as particles, which prompted Zabusky and Kruskal to introduce the term “soliton”^1^. Some media, such as nematic liquid crystals, offer especially rich opportunities for solitary waves and solitons studies.

A uniaxial nematic is a nonpolar fluid with a long-range orientational order of molecules specified by a director $\widehat {\bf{n}}$, with the properties $\widehat {\bf{n}} \equiv - \widehat {\bf{n}}$ and $\left| {\widehat {\bf{n}}} \right|^2 = 1$. The orientational order makes the nematic fluid anisotropic, with a strong coupling between $\widehat {\bf{n}}$ and fluid velocity; this order also enables nonlinear response to external fields. For many decades, research focused on the response to the electric field and resulted in revolutionary development of liquid crystal displays. An elementary unit, or pixel, of a typical display represents a sandwich cell with a nematic fluid confined between two glass plates with transparent electrodes. When a static or low-frequency (<10^3^ kHz) electric field **E** is applied across the cell, it induces collective reorientation of the nematic molecules. The mechanisms are rooted in the anisotropy of nematic properties such as dielectric permittivity and electric conductivity: permittivity and conductivity measured along $\widehat {\bf{n}}$ are different from those measured perpendicularly to $\widehat {\bf{n}}$. The reorientation of the director $\widehat {\bf{n}}$ is either homogeneous over the area of electrodes, as in most liquid crystal displays^2^, or periodically modulated, as in the phenomenon called electroconvection^2,3^, similar to Rayleigh–Bénard thermal convection^4^.

Early studies of spatially restricted director perturbations, termed as solitons or, more properly, topological configurations^5^, in nematics started about 50 years ago with the discussion of static linear and planar solitons produced by the magnetic or electric fields^6^. These solitons form when the external field aligns the director parallel to itself because of dielectric or diamagnetic anisotropy. Since the nematic is nonpolar, the states $\widehat {\bf{n}}$ and $- \widehat {\bf{n}}$ that are both parallel to the applied field are equivalent to each other. If both states are trapped by the applied field, there must be a transition region in which $\widehat {\bf{n}}$ reorients by *π*. This region of a smooth director reorientation of a finite width (determined by a balance of elasticity and coupling to the field) represents a static linear or planar topological soliton^5^. Multidimensional static solitons such as three-dimensional (3D) particle-like nonsingular perturbations of the director field $\widehat {\bf{n}}\left( {\bf{r}} \right)$ are deemed unstable with respect to shrinking. The reason is that a decrease in size of a 3D soliton, *L* → *μL* by a factor *μ* < 1 entails a decrease in the total elastic energy, *F* → *μF*^5^. Static 3D topological solitons can be stable in cholesterics, which are chiral nematics with helical twisting of the director. These formations, observed by many research groups^7–16^, are static and do not require any motion (although they can be forced to move by an external field^12^). Their stability against shrinking is topologically protected and guaranteed by the helicoidal structure of the cholesteric that maintains a fixed pitch. Regions of $\widehat {\bf{n}}$ and $- \widehat {\bf{n}}$ that differ by a director twist by *π* are separated by half of the pitch and cannot be moved closer to each other without a dramatic increase of the elastic energy^10,17,18^. To the best of our knowledge, there have been no reports on dynamic 3D particle-like solitons in liquid crystals.

Current studies on solitons in nematics mostly deal with optical solitons^19–21^ representing propagating self-focused laser beams called “nematicons”^19^. Studies of nematicons are a part of a very broad research on optical solitons in different nonlinear media^22–27^, see reviews by Malomed et al.^28,29^. Optical solitons are usually described using a nomenclature (*m* + 1)D, which means that the light beam can diffract in *m* dimensions as it propagates in one dimension^22,28^. Of especial interest are the so-called “light bullets”^30^ or (3 + 1)D spatiotemporal solitons, which are self-confined in the longitudinal and transverse directions and can be used in fast optic-logic systems^31^. Multidimensional solitons, unlike their 1D counterparts, are vulnerable to various instabilities and are extremely hard to realize experimentally, see the discussion in refs.^27–29^. For example, there are no reports on experimental observations of stable light bullets that can survive collisions without losing energy.

In this work, we present an experimental realization of “director bullets”, representing 3D solitary waves with dual particle–wave character that propagate through a slab of a uniformly aligned nematic liquid crystal, being powered by an alternating current (AC) electric field. The bullet is a nonsingular bow-like perturbation of the director from the uniform state. It is localized along all three spatial dimensions and does not spread while moving over macroscopic distances thousands times longer than their size. Within a bullet, the director perturbation oscillates with the frequency of the applied AC electric field and breaks the fore-aft symmetry, which results in rapid propagation perpendicularly to the initial alignment direction. Director bullets survive collisions with each other, restoring shape and constant velocity, as true solitons^1^. They show short-range attractive and repulsive interactions. The bullets are topologically equivalent to a uniform state (i.e., the director field can be smoothly transformed into a uniform state) and have no static analogs.

## Results

### Experimental design

**Fig. 1:**
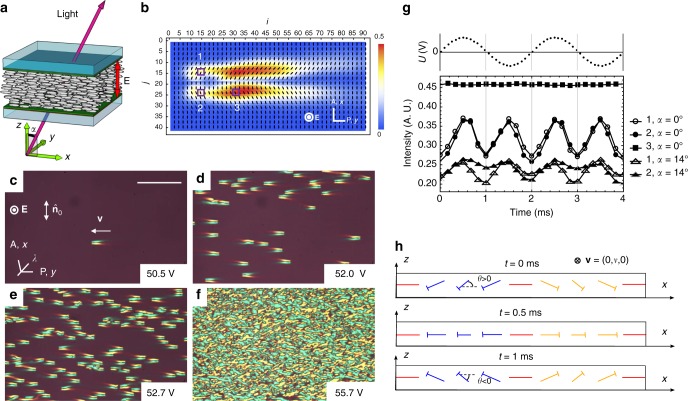
Director bullets in a planar nematic cell. **a** Cell scheme. **b** Transmitted light intensity map and director distortions in the *xy* plane within a single bullet (*U* = 65.4 V, *f* = 500 Hz, *T* = 40 °C, *d* = 8.0 μm). The bar shows a linear scale of transmitted light intensity; the pixels are labeled by (*i*, *j*) coordinates. The length of a single step, *i* → *i* ± 1 or *j* → *j* ± 1, is 1 μm. **c**–**e** Director bullets are observed between crossed polarizers and a compensator with the optic axis *λ* as the voltage *U* increases as indicated (*f* = 450 Hz, *T* = 45 °C, *d* = 7.5 μm). Scale bar 200 μm. **f** Irregular stripe domains formed under a high applied voltage *U* = 55.7 V. **g** Dynamics of the transmitted light intensity for the same parameters as in **b**, at locations 1, 2, and 3 indicated in **b** for normal, *α* = 0 and oblique, *α* = 14° incidence of light; *α* is measured in the *xz* plane. **h** Schematic 3D director field at the head of the bullet for three instants separated by a quarter period of the applied voltage. The nails represent a tilted director, with the heads closer to the observer than the ends

We use a nematic 4′-butyl-4-heptyl-bicyclohexyl-4-carbonitrile (CCN-47) with a negative anisotropy of permittivity, Δ*ε* = *ε*_∥_ − *ε*_⊥_ < 0 and conductivity, Δ*σ* = *σ*_∥_ − *σ*_⊥_ < 0^32^; the subscripts indicate whether the component is measured along $\widehat {\bf{n}}$ or perpendicularly to it. The nematic is aligned uniformly, $\widehat {\bf{n}}_0$ = (1, 0, 0), in flat cells of thickness *d* = (3–30) μm, Fig. 1a. A sinusoidal field **E** = (0, 0, *E*) of frequency *f* = 20 Hz–5 kHz is perpendicular to the *xy* plane of the cell. This combination of material parameters Δ*ε* < 0 and Δ*σ* < 0, and field/director geometry are chosen to avoid two principal mechanisms of field-induced director reorientation. Namely, the condition Δ*ε* < 0 implies that dielectric anisotropy favors the initial orthogonal orientation of $\widehat {\bf{n}}$ with respect to **E**; the condition Δ*σ* < 0 rules out the electrohydrodynamic Carr–Helfrich director instability^2,3^. Although the two principal mechanisms are eliminated, the experiment clearly demonstrates field-induced formation and propagation of localized solitary waves of the director, as detailed below.

### Nucleation and structure of solitons

As voltage increases above some frequency-dependent threshold *U*_soliton_, 3D localized director distortions nucleate and move perpendicularly to $\widehat {\bf{n}}_0$ and **E**, over distances much longer than their size, without spreading and surviving collisions, thus representing solitons, Fig. 1b–e and Supplementary Movies 1 and 2. Viewed under a polarizing microscope with one of the polarizers along $\widehat {\bf{n}}_0$, the moving solitary wave, which we call a director bullet, resembles a “flying tuxedo”, because of the characteristic head–tail asymmetry, Fig. 1b–e. Outside the bullet, $\widehat {\bf{n}}$ remains parallel to the *x*-axis, and the intensity of transmitted light is close to 0. Inside the bullet, transmission increases, indicating azimuthal (within the *xy* plane) deviations of $\widehat {\bf{n}}$ from the initial alignment along the *x*-axis, $\widehat {\bf{n}}_0$ = (1, 0, 0), by some angle *φ* ≈ 20°–35°, Fig. 1b–e. When a red plate compensator (530 nm) is inserted with the optic axis *λ* making 45° with the polarizer, one shoulder of the bullet appears yellow and the other blue, Fig. 1c–e. The reason is that the local retardance of the nematic, associated with the optical axis $\widehat {\bf{n}}$, can either add to or subtract from the optical retardance of the compensator^5^. Namely, in the blue region (higher order interference colors), $\widehat {\bf{n}}$ tilts toward *λ*, while in the yellow region (lower order interference colors), $\widehat {\bf{n}}$ tilts away from *λ*^5^, compare Fig. 1b–e. The in-plane director perturbation within the bullet resembles a bow, Fig. 1b.

**Fig. 2:**
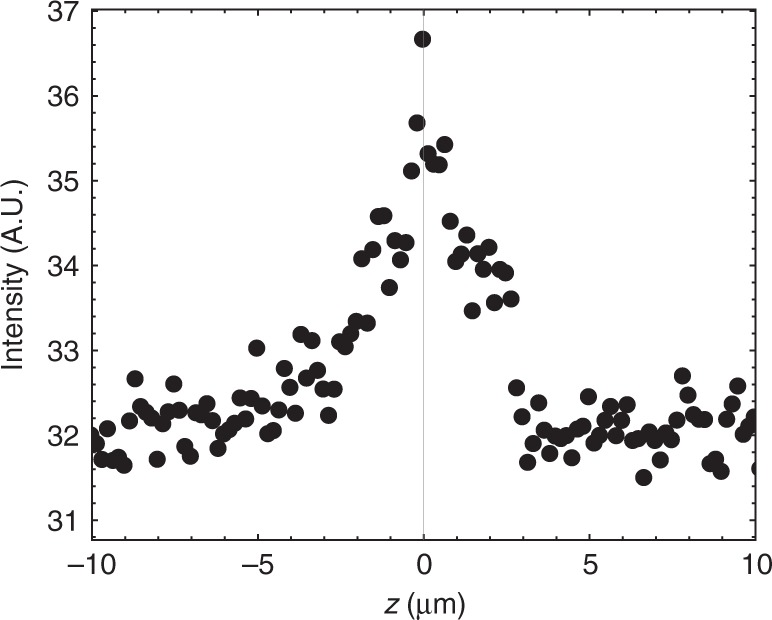
Fluorescent signal measured across the cell in the presence of bullets. Nematic cell under an AC field, *U* = 42.0 V, *f* = 200 Hz. Probing light polarized along the *y*-axis. The background fluorescence signal from a cell at zero voltage is subtracted from the plot. *d* = 8.0 µm, *T* = 35 °C. The maximum intensity observed at the middle plane, *z* = 0, indicates that the bullets propagate through the middle of the nematic slab

Fluorescence confocal polarizing microscopy (FCPM) shows that the azimuthal angle reaches its maximum value *φ* ≈ 20°–35° in the middle plane of the cell, *z* = 0, Fig. 2. As one moves toward the top and bottom boundaries of the nematic slab, *z* = ±*d*/2, the azimuthal angle diminishes as the in-plane surface anchoring keeps the actual $\widehat {\bf{n}}$ close to the initial director, $\widehat {\bf{n}}_0$. This result implies that the bullets are propagating through the middle of the nematic slab.

**Fig. 3:**
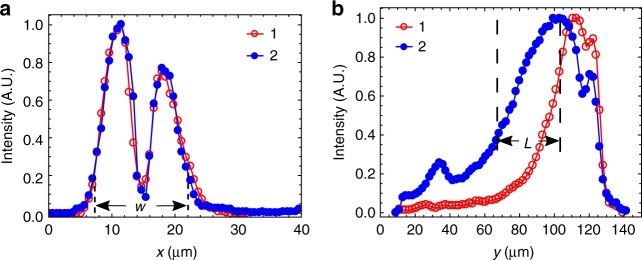
Width and length of bullets. **a** Transmitted light intensity measured across two bullets, “1” (*f* = 500 Hz, *U* = 41.2 V) and “2” (850 Hz, 56.4 V) in the same cell with *d* = 7.8 µm; the profile is measured as a function of *x*-coordinate along the line that passes through the transmission maximum (such as line *i* = 33 in Fig. 1b). **b** Longitudinal profiles of the same 1 and 2 bullets, measured as a function of *y*-coordinate along the line that passes through maximum transmittance (such as line *j* = 14 in Fig. 1b). Note that the width *w* shows practically no dependence on the applied voltage, while the length *L* increases with voltage

At a fixed cell thickness *d*, the solitons preserve their width, *w* ≈ 2*d*, along the *x*-axis; *w* does not change with *f* and voltage *U*, Figs. 1c–e and 3a. Note here that *w* characterizes the width of the bullet’s head, in the region where the azimuthal deformations are the strongest; in Fig. 1b, this region corresponds to *i* = 33. The width *w* is determined from the image of the bullet taken between crossed polarizers and a compensator, measured between the two points at which the intensity of transmitted light drops from its maximum to 20% of the maximum, Fig. 3a. Toward the bullet’s tail, the director gradually relaxes to the uniform state and the width becomes larger than *w*, by about 30%, Fig. 1c–e. The length *L* of the bullets, defined as the distance between the point of maximum light intensity and a point toward the tail where the transmitted intensity is smaller by a factor *e* ≈ 2.7, is in a range *L* ≈ (20–50) μm and increases with *U*, Fig. 3b. Both *w* and *L* are orders of magnitude smaller than the system's lateral extensions of 5 mm.

The director in the left and right sides of a soliton synchronously tilts up and down by an angle *θ* (measured between $\widehat {\bf{n}}$ and the *xy* plane), thus causing periodic modulations of light transmission, Fig. 1g. The oscillations of *θ* are localized at the head of the tuxedo, coordinates *i* = 7–25 in Fig. 1b, with the maximum |*θ*| ≈ 20°–35° in the pixels labeled 1 and 2 in Fig. 1b. In the rest of the tuxedo, *i* > 25 and pixel 3 in Fig. 1b, the light intensity does not oscillate, thus *θ* = 0, Fig. 1g.

**Fig. 4:**
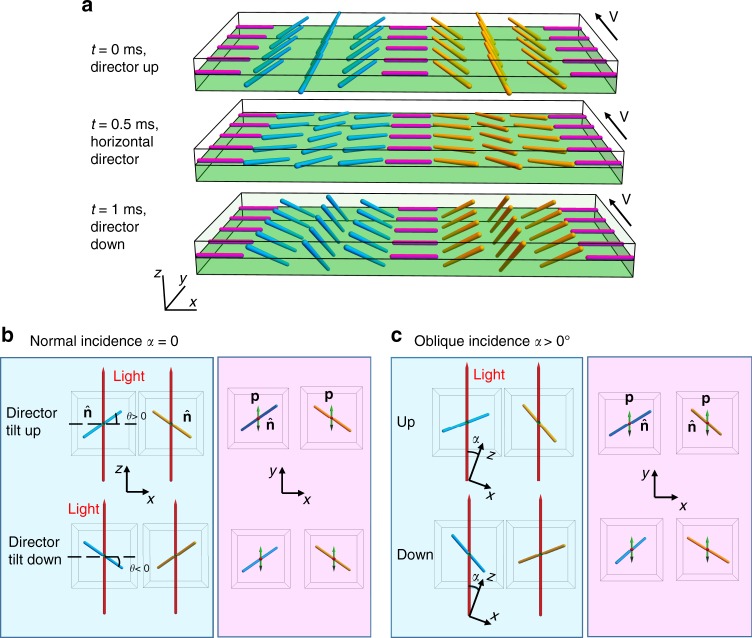
Polar tilts of the director within the head of the soliton. **a** Scheme of the director. **b** Normal incidence of the light beam polarized along the *y*-axis; left and right parts of the soliton produce the same angle between light polarization (**p**) and the local director. **c** oblique incidence produces a different light transmittance through the left and right sides of the soliton because the angle between the local director and **p** is different in these two sides

To characterize the period and polarity of oscillations, we used oblique incidence, Fig. 4, with an angle *α* ≈ 14° measured in the incidence *xz* plane between the probing light beam and the *z*-axis, Fig. 1a. This value of *α* represents the maximum tilt of a cell for a given distance to the microscope’s objective; smaller *α*’s yield a weaker contrast between the left and right sides of the tuxedo. For oblique incidence, the up and down tilts of the director, Fig. 4a, produce different projections onto the polarization direction of light (the *y*-axis), Fig. 4b, c. The light intensity variations for pixels 1 and 2 in Fig. 1g show that *θ* oscillates with the same frequency *f* as the applied field, Fig. 1h. There is a phase shift (about *π*/2) between *E* and *θ*. Namely, when the field is zero, the light intensity reaches a minimum, Fig. 1g, which implies that |*θ*| reaches its maximum. The oscillations of *θ* do not change substantially the azimuthal angle, *φ*, in the coordinate frame moving with the soliton.

**Fig. 5:**
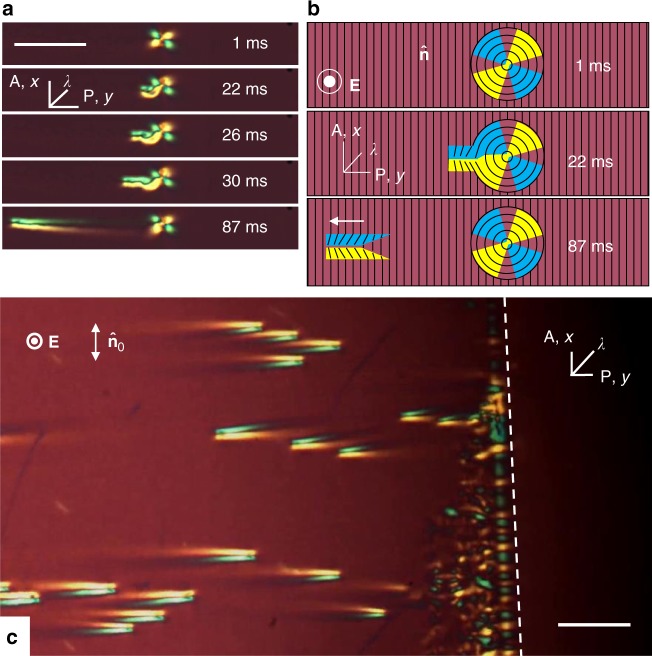
Nucleation of solitons at irregularities. **a** Nucleation in a dust particle that creates a circular director field around itself, five instants of time are shown; *U* = 87.4 V, *f* = 1000 Hz, *T* = 45 °C, and *d* = 8.2 μm. Scale bar 100 µm. **b** Corresponding qualitative in-plane director scheme of nucleation in the dust particle. **c** Electrode edge (marked by a dashed line) absorbs and nucleates solitons; *U* = 72.2 V, *f* = 1000 Hz, *T* = 50 °C, *d* = 8.0 μm. Scale bar 50 µm

The solitary waves nucleate at irregularities such as surface imperfections, dust particles, Fig. 5a, b, and at edges of the electrodes, Fig. 5c (Supplementary Movies 3 and 4). Some of these nucleation sites, representing dust particles, are clearly visible under the microscope, since they distort the director field around them even in the absence of the applied voltage, Fig. 5a, b. When the field is applied, these distortions produce bullets. However, very often, the bullets are formed at sites that are invisible under an optical microscope. These invisible sites must be associated with very small dust particles or low-contrast surface alignment imperfections, since they produce multiple bullets in sequence. If the field is switched off and then on, these sites are most likely to start producing bullets again. Microscopic mechanism of bullet generation requires further studies.

**Fig. 6:**
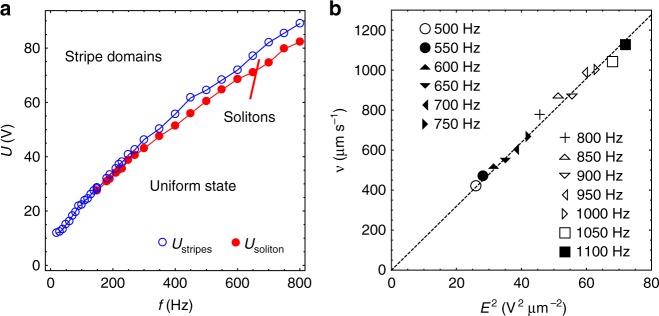
Soliton properties as a function of the applied field. **a** Frequency dependencies of *U*_soliton_ and *U*_stripes_ (8.0 μm, 45 °C). **b** Velocity of solitons vs. square of the electric field (7.8 μm, 50 °C); the applied field corresponds to the frequencies specified in the legend

The propagating director bullets exist in a broad range of thicknesses, *d* = (3.0–19.5) μm, frequency, *f *≈ (0.1–4) kHz, and temperature, *T* = (30–58) °C. The frequency range is limited from below by *f*_soliton_ that decreases as *d* increases. At 45 °C, *f*_soliton_ = 550 Hz and 130 Hz in cells with *d* = 3.5 μm and 9.3 μm, respectively. The most plausible reason for the absence of solitons at *f* < *f*_soliton_ is the presence of ions that move toward the electrodes and screen the applied electric field. At a fixed *f* > *f*_soliton_, the solitary waves exist in a narrow range of voltages *U* = (1–1.1)*U*_soliton_, Fig. 6a. Existence of a nonzero *U*_soliton_ can be explained by the elastic resistance of the nematic to director distortions created by the electric field. The number of bullets increase with *U*, Fig. 1c–e. Once the voltage exceeds another threshold, *U*_stripes_ ≈ 1.1*U*_soliton_, the solitons are replaced by an irregular array of stripes, Fig. 1f. Both *U*_soliton_ and *U*_stripes_ increase with *f*, Fig. 6a. We relate this increase with the fact that the nematic response is polar: higher frequencies of excitation require a higher amplitudes of the field to develop sufficient director deformations during one-half period of the field. In thick cells, *d* > 19.5 μm and low frequencies, *f* < *f*_soliton_, the uniform state changes directly to the stripe pattern. Note that it is presently not clear what might be the highest frequency of bullet generation, since voltage increases with frequency and experimental cells are often short-cut at frequencies above 1 kHz.

The velocity **v** of solitons, directed along the *y*-axis, is of a huge amplitude, *v* ≈ (10–50) *L* s^−1^, and grows as *v* = *βE*^2^, where *β* ≈ 1.5 × 10^−17^ m^3^ V^−2^ s^−1^, Fig. 6b. This dependence is indirect, since for a fixed *f*, the solitons exist only within a narrow range of voltages. Figure 6b shows data for different frequencies. Note that the value of the electric field alone is not sufficient to characterize the production of bullets; one needs to specify both the voltage and the cell thickness, since, for example, the bullets do not form in slabs thicker than *d* = 19.5 μm, no matter what is the field.

### Collisions and interactions of solitons

**Fig. 7:**
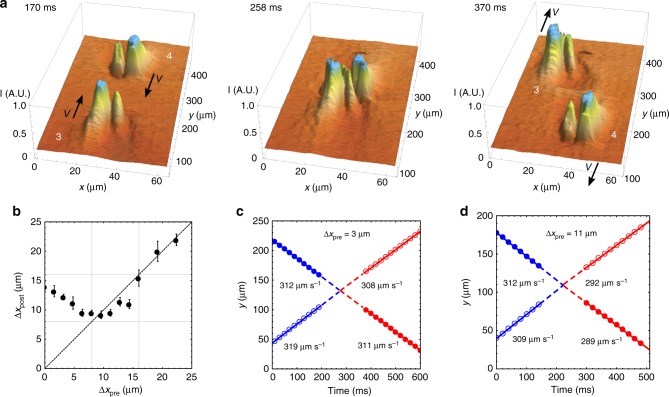
Collisions and interactions of solitons. **a** 3D plots of transmitted light intensity through solitons “3” and “4” vs. *x*, *y* coordinates before, during, and after collision; Δ*x*_pre_ < *w*/2; collision results in repulsion, Δ*x*_post_ > Δ*x*_pre_ (*U* = 55.2 V, *f* = 800 Hz, *T* = 50 °C, *d* = 8.0 μm). **b** Post-collision separation Δ*x*_post_ as a function of the impact factor Δ*x*_pre_ (*U* = 54.8 V, *f* = 400 Hz, *T* = 45 °C, *d* = 8.0 μm). **c** Time dependence of the *y*-coordinates of two colliding solitons, with Δ*x*_pre_ < *w*/2; *U* = 52.1 V, *f* = 450 Hz, *T* = 45 °C, *d* = 7.5 μm. **d** The same dependence for solitons with *w*/2 < Δ*x*_pre_ < *w*; the same experimental conditions as in **c**. The lines represent linear fitting used to calculate the velocities indicated in the figures

**Fig. 8:**
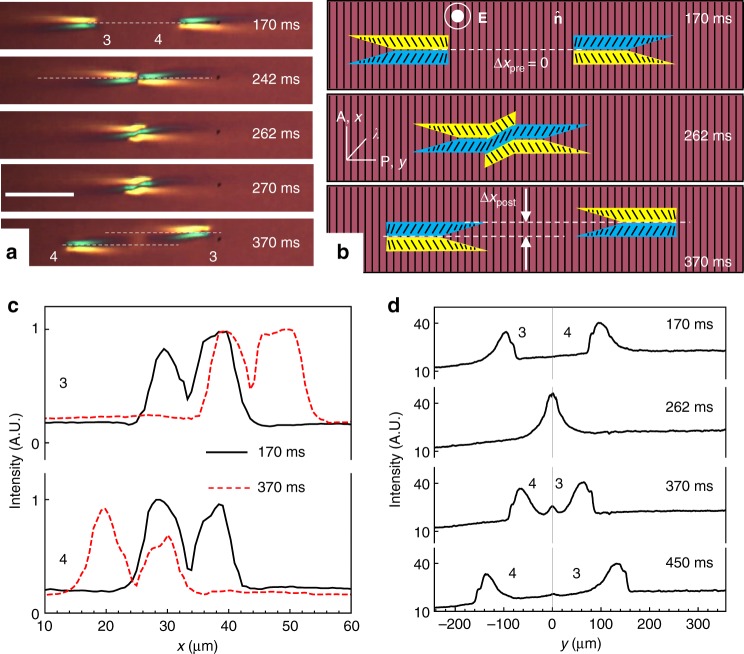
Collision and recurrence of solitons. **a**. Polarizing microscopy textures of collision of solitons “3” and “4”; Δ*x*_pre_ < *w*/2; *U* = 55.2 V, *f* = 800 Hz, *T* = 50 °C, *d* = 8.0 μm; scale bar 100 µm; the same solitons are also shown in Fig. 7a. **b** Corresponding in-plane director schemes. **c** Transmitted light intensity for solitons as a function of *x*-coordinate before and after collision (measured along the line *i* = 15 in Fig. 1b). The left and right sides are of different intensity because of the presence of the red plate that imparts the blue and yellow interference colors on the two sides of the soliton. **d** Profiles of the solitons as a function of *y*-coordinate before and after collision, obtained by integrating light intensity transmitted through the soliton width (along the *x*-axis)

Bullets moving toward each other can collide, Fig. 7. As Fig. 2 suggests, bullets move in the middle of the cell, close to *z* = 0. Since the width of the bullet is twice the cell thickness, *w* ≈ 2*d*, bullet-collision outcomes depend mostly on their locations in the *xy* plane. In this plane, we define the impact parameter, Δ*x*_pre_, which is the distance between the centers of two bullets moving toward each other measured along the *x*-axis. The most frequent scenario is that the bullets reshape during the collision and then recover their structure and constant velocity, which might be different from the pre-collision velocity, Figs. 7 and 8, and Supplementary Movie 5. The post-collision recovery demonstrates the particle-like soliton character of the bullets. They also show short-range interactions that depend on Δ*x*_pre_. When Δ*x*_pre_ > *w*, solitons pass each other without noticeable perturbations. Solitons that collide “head-to-head”, Δ*x*_pre_ < *w*/2, Figs. 7a and 8a, show a repulsion along the *x*-axis, as their post-collision separation increases, Δ*x*_post_ > Δ*x*_pre_, Figs. 7b and 8b. Solitons with *w*/2 < Δ*x*_pre_ < *w* attract each other along the *x*-axis, as Δ*x*_post_ < Δ*x*_pre_, Fig. 7b. The solitons completely recover their shape after collisions, Figs. 7a and 8. Figure 7c, d presents trajectories of soliton pairs with Δ*x*_pre_ < *w*/2 and *w*/2 < Δ*x*_pre_ < *w*, respectively. The solitons show constant pre- and post-collision velocities.

Figure 8a presents the polarizing microscope texture of the same soliton pair as in Fig. 7a in order to clarify director reconstruction in the *xy* plane during head-to-head collision, Fig. 8b. Director distortions along the *x*-axis in the overlapping zone (clearly seen at the moment of time 262 ms in Fig. 8b) cause elastic repulsion of the solitons, which results in their separation along the *x*-axis. Post-collision reconstruction of the solitons’ shape is evidenced by the profiles of transmitted light intensity measured across the solitons, Fig. 8c, and along the solitons, Fig. 8d. In Fig. 8d, the light intensity is integrated over the *x*-axis in order to avoid artefacts associated with the repulsion of solitons along this axis.

**Fig. 9:**
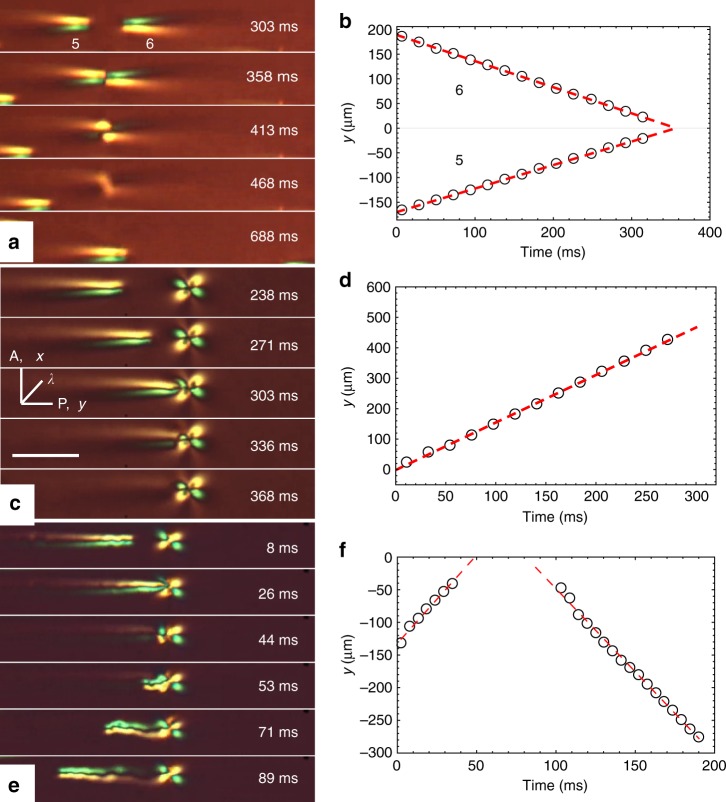
Scenarios of soliton's interactions with each other and with obstacles. **a** Polarizing microscopy of time sequence of annihilation of solitons “5” and “6”; *U* = 45.1 V, *f* = 600 Hz, *T* = 50 °C, *d* = 8.0 μm. **b** Locations of annihilating solitons (determined as the coordinate of the maximum of light transmittance) vs. time. **c**, **d** Disappearance of a soliton at a dust particle. *U* = 65.6 V, *f* = 800 Hz, *T* = 50 °C, *d* = 7.7 μm. Scale bar 100 µm. **e**, **f** Reflection of a soliton on a dust particle. *U* = 87.4 V, *f* = 1000 Hz, *T* = 45 °C, *d* = 8.2 µm. **a**, **c**, **e** present polarizing microscopy textures under crossed polarizers and a compensator, as shown in **c**

Other outcomes of pair collisions include annihilation, Fig. 9a, b (Supplementary Movie 6), and disappearance of one soliton. Collision with an irregularity such as a dust particle can result in soliton’s death, Fig. 9c, d (Supplementary Movie 7) or reflection, Fig. 9e, f (Supplementary Movie 8).

## Discussion

The described electrically driven dynamic particle-like soliton, or the director bullet, represents a propagating solitary wave of director deformation in a nematic liquid crystal slab that is self-trapped along all three spatial coordinates. From the point of view of classification used to describe optical solitons, the director bullets can be called (2+1+1)D solitons, where “2” refers to the transversal (*x*-axis) and longitudinal (*y*-axis) self-confinement; the first “1” refers to transversal surface anchoring-imposed confinement along the *z*-axis, the second “1” specifies unidirectional propagation along the *y*-axis.

The solitons preserve their shape during rapid motion over long distances and after collisions. Their interactions involve short-range attraction and repulsion. The ability to propel along the *y*-axis is caused by two factors: (a) up and down tilts of the director at the head of soliton with the frequency of the applied AC electric field and (b) broken fore-aft symmetry of the director structure, in which the sign of the azimuthal angle *φ* does not change when the polar angle *θ* oscillates around zero.

The electrically induced solitons represent a unique electrohydrodynamic effect in nematics. The observation is distinct in two aspects. First, although the electroconvection patterns have been observed by many groups^3^, there were no reports of self-trapped dynamic soliton waves. The closest structures are “worms”^33^, representing elongated formations parallel to the initial director $\widehat {\bf{n}}_{\mathrm{0}}$ = (1, 0, 0) with finite width, but no well-defined length. Another localized structures are islands filled with stripe domains^34^. The islands grow upon the voltage increase till they fill the entire area with stripe pattern; they do not move laterally. Both worms and islands occur in nematics with Δ*ε* < 0 and Δ*σ* > 0^35^. This combination fits the classic Carr–Helfrich mechanism^2^ of electroconvection, according to which the positive anisotropy of conductivity is responsible for destabilization of the planar state. A spatial director fluctuation in the presence of an electric field produces space charges and corresponding Coulomb forces which cause instability, usually in the form of space-filling periodic stripes.

The second aspect is that the solitons are observed in the so-called (−, −) nematic with Δ*ε* < 0 and Δ*σ* < 0. The Carr–Helfrich mechanism is irrelevant for (−, −) nematics, as dielectric and conductivity torques could only stabilize the planar state. There are two other mechanisms of coupling between the electric field and the director: surface polarization and flexoelectric polarization. Surface polarization can occur at the boundaries of nematic cells because of preferred alignment of molecular dipoles. In the presence of the electric field, it can cause surface deformations^36^, but not the bulk deformations, which contradicts the observed properties of the solitons with maximum tilts in the middle of the cell. Surface polarization as a reason for solitons is thus ruled out. The main mechanism of soliton formation should be related to flexoelectric polarization.

Flexopolarization, ${\bf{P}}_{{\mathrm{fl}}} = e_1\widehat {\bf{n}}{\mathrm{div}}\widehat {\bf{n}} - e_3\widehat {\bf{n}} \times {\mathrm{curl}}\widehat {\bf{n}}$, where *e*_1_ and *e*_3_ are the flexoelectric coefficients^5^, occurs whenever the director contains splay and bend distortions, which is the case of the director bullets, Fig. 1b, h. Flexopolarization leads to spatial charge density *ρ*_fl_ = ∇ · **P**_fl_ and a corresponding Coulomb force *ρ*_fl_**E**^37^. Flexopolarization was suggested as a prime mechanism of the so-called “nonstandard” stripe electroconvection in (−, −) nematics^38^, with textures similar to irregular stripes in Fig. 1f. There is only one instance of localized formations in (−, −) nematics, in the shape of “butterflies” that could move in the plane of the cell, as observed by Brand et al.^39^. The report^39^, however, did not reveal the director structure of formations, nor the direction of motion.

The periodic oscillation of the soliton’s director with the frequency *f*, Fig. 1g, h, offers a strong support of the idea that the flexoelectric torque, **Γ**_fl_ = **P**_fl_ × **E**, is responsible for bullet formation, since it is linear in the field. Further support is provided by the similar symmetry properties of flexoelectric stripes described by Krekhov et al.^37^ and our solitons. Namely, the reversal of the electric field polarity causes reversal of the polar angle *θ,* but leaves intact the sign of *φ*. The theory^37^ predicts the same type of frequency dependence of *U*_stripes_ as in Fig. 2a. The flexoelectric force driving a soliton can be estimated roughly as e^*^U ~ 5 × 10^−10^ N, where e^*^ ~ 10^−11^ C m^−1^(ref.^2^) is an effective flexocoefficient and *U* = 50 V. This estimate agrees very well with the expected viscous drag force, ~ *Rηv* ~ 5 × 10^−10^ N, where *R* ~ 10 μm is the effective radius of the soliton, *η* ≈ 60 mPa s is the viscosity of CCN-47, and *v* ≈ 0.8 × 10^−3^ m s^−1^ that corresponds to *U* = 50 V, Fig. 6b.

The director bullets are not unique to CCN-47. We observe them also in other materials, such as ZLI-2806 (Merck). Maria Helena Godinho and Charles Rosenblatt observed similar formations in their nematic systems (unpublished results, private communications). A critical condition for the soliton formation is relatively low conductivity. In the studied CCN-47, *σ*_||_, *σ*_⊥_ ≈ (5–6) × 10^−9^ Ω^−1^ m^−1^, which is much smaller than the conductivity, 10^−7^ Ω^−1^ m^−1^, is usually reported in the studies of stripe electroconvective patterns in (−, −) nematics^40^. When the conductivity of CCN-47 is increased to 10^−7^ Ω^−1^ m^−1^ and the concentration of ions is raised above 10^21^ m^−3^ by the addition of salts such as tetrabuthylammonium bromide, the solitons are suppressed and the nematic undergoes a direct transition to stripe domains. The condition of low conductivity might explain why the soliton director bullets have not been reported before.

To summarize, we demonstrated that electric field can cause a unique structural response of a nematic liquid crystal representing propagating solitary waves that are trapped along all three spatial dimensions. The dynamics of these director bullets are caused by periodic oscillations of the director with the same frequency as the frequency of the electric field which suggests that the underlying mechanism is related to flexoelectric polarization. The presented rich dynamic behavior of 3D particle-like solitons in a system that is relatively easy to control should open the door to a broad range of further studies. Of especial importance, would be the study of director deformations during pair-wise collisions, dependency of the soliton properties on the control parameters, and collective behavior of solitons at concentrations that are sufficiently high for multiparticle interactions. The observed solitons pose a formidable problem for a detailed theoretical description that should take into account a complex mix of dielectric, conductive, flexoelectric, elastic, and anisotropic viscous forces.

## Methods

### Materials

The studied nematic is a single-component 4′-butyl-4-heptyl-bicyclohexyl-4-carbonitrile, abbreviated as CCN-47 (Nematel GmbH). The phase diagram of CCN-47 is smectic A 29.9 °C nematic 58.5 °C isotropic phase. We confirmed the conclusion of Dhara and Madhusudana^32^ that the anisotropy of both the permittivity and conductivity of CCN-47 is negative, by performing independent measurements using an LCR meter 4284A (Hewlett-Packard), and cells with planar (alignment agent polyimide PI-2555, HD MicroSystems) and homeotropic (polyimide SE1211) alignment. In particular, at 45 °C and 4 kHz, *ε*_⊥_ ≈ 8.8, *ε*_∥_ ≈ 4.6, *σ*_⊥_ ≈ 6.1 × 10^−9^ Ω^−1^ m^−1^, *σ*_||_ ≈ 4.9 × 10^−9^ Ω^−1^ m^−1^ and concentration of ions is 4 × 10^20^ m^−3^. The effective viscosities of CCN- 47 are *η*_||_ = 57 mPa s and *η*_⊥_ = 58 mPa s for a motion parallel and perpendicular to the director, respectively, as determined by tracking Brownian motion of polystyrene colloidal spheres of diameter 5 μm and tangential surface anchoring^41^.

### Generation of solitons

The cells were aligned in a planar fashion by using rubbed layers of PI-2555. The directions of rubbing at two opposite plates are antiparallel. The glass plates contain transparent indium tin oxide electrodes of area 5 × 5 mm^2^. The temperature of the cell is controlled with a Linkam LTS350 hot stage and a Linkam TMS94 controller. The AC electric field was applied using a waveform generator (Stanford Research Systems, Model DS345) and amplifier (Krohn-hite Corporation, Model 7602).

### Optical characterization of solitons

The material is of low birefringence, Δ*n* = *n*_e_ − *n*_o_ ≈ 0.03, ref. ^32^ (*n*_e_ and *n*_o_ are the extraordinary and ordinary refraction indices, respectively), which makes it convenient to explore the solitons and the associated director deformations using standard polarizing microscopy with a wave-plate (red plate) optical compensator and FCPM^42^. The director distortions are characterized by analyzing the transmitted light intensity, *I* = $I_0{\kern 1pt} {\mathrm{sin}}^2\left( {2\varphi - 2\psi } \right) {\mathrm{sin}}^2\left( {{{{\noexpand\iGamma} /}}2} \right)$, determined by optical phase retardance,

*Γ* = $\frac{{2\pi {\kern 1pt} {\mathrm{\Delta }}n{\kern 1pt} d}}{\lambda }$ $\left( {1 - {\mathrm{sin}}^2\theta {\kern 1pt} {\mathrm{cos}}^2\varphi - \frac{{{\mathrm{sin}}{\kern 1pt} \alpha {\kern 1pt} {\mathrm{sin}}{\kern 1pt} 2\theta {\kern 1pt} {\mathrm{cos}}{\kern 1pt} \varphi }}{{\bar n}}} \right.$ − $\left. {\frac{{{\mathrm{sin}}^2\alpha \left( {{\mathrm{sin}}^{\mathrm{2}}\theta {\kern 1pt} {\mathrm{cos}}^2\varphi - {\mathrm{cos}}{\kern 1pt} 2\theta } \right)}}{{\bar n^2}}} \right)$, and the angle *ψ* between *y*-axis and the polarizer; here, $\bar n$ = (*n*_e_ + *n*_o_)/2 is the average refractive index. When the director is along the polarizer or the analyzer, the texture is dark. The azimuthal angle *φ* is determined by rotating the cell in the normal incidence geometry (*α* = 0) around the *z*-axis (scanning *ψ*) and measuring the corresponding change in the transmitted light intensity. The value of *φ* corresponds to a rotating angle of the cell at which one side of the tuxedo is extinct. Note that the Mauguin effect^2^ of adiabatic, following light polarization by the director twist, does not produce a strong modification of the measured *φ* because of low birefringence of CCN-47 and because we use high-magnification objectives (20x and 40x) with high numerical aperture and oblique propagation of light. Knowing *φ*, we calculate the maximum polar tilt *θ*_max_ from the minimum light transmittance measured as a function of time, using the formula above.

We used polarizing Nikon TE2000 inverted microscope equipped with two cameras: emergent HR20000, with resolution of 5120 × 3840 pixels and the frame rate up to 1000 frames per second, and MotionBLITZ EOSens mini1 (Mikrotron GmbH), with the frame rate up to 6000 frames per second. The y-coordinate of a soliton is defined as the location of the maximum light intensity transmitted through the soliton observed between the two polarizers and an optical compensator; the transmittance maps are analyzed using Mathematica 11.2. Measuring the *y*-coordinate of the soliton as a function of time and fitting it with a linear dependency yields the velocity.

To verify that the maximum director distortions in the bullets are located in the middle of a cell, we used fluorescence confocal polarizing microscope (FCPM) based on Olympus Fluoview BX-50^43^. CCN-47 is doped with a small amount (0.1 wt%) of fluorescent dye *n*,*n*′-bis(2,5-di-tert-butylphenyl)-3,4,9,10-perylenedicarboximide (BTBP), purchased from Molecular Probes. BTBP molecules are elongated and align parallel to the director. FCPM uses a linearly polarized laser beam to probe the specimen under normal incidence. The fluorescent signal is maximum when the light polarizations are parallel to the transition dipole of BTBP and thus to the director^43^, and minimum when the director and light polarization are orthogonal. The scanning focused laser beam is linearly polarized along the *y*-axis. In the absence of the electric field, there are no solitons. Since the initial director $\widehat {\bf{n}}_0$ is perpendicular to the direction of light polarization, the fluorescent signal from the cell is very weak (being caused mostly by director fluctuations). When the field is on and the dynamic solitons with a nonzero *y*-component of the director are generated, the fluorescent signal in the middle of the cell increases noticeably, which demonstrates that the maximum of director deviation from the *x*-axis occurs in the middle plane of the cell. Figure 2 shows the difference in the fluorescent signals of a cell with the bullets and a uniform cell with a clear maximum in the middle of the cell.

### Data availability

The data that support the findings of the study are available from the corresponding author upon reasonable request.

## Electronic supplementary material

Description of Additional Supplementary Files

Supplementary Movie 1

Supplementary Movie 2

Supplementary Movie 3

Supplementary Movie 4

Supplementary Movie 5

Supplementary Movie 6

Supplementary Movie 7

Supplementary Movie 8
