# Publisher Correction: Electrically driven three-dimensional solitary waves as director bullets in nematic liquid crystals

**DOI:** 10.1038/s41467-018-06028-0

**Published:** 2018-08-30

**Authors:** Bing-Xiang Li, Volodymyr Borshch, Rui-Lin Xiao, Sathyanarayana Paladugu, Taras Turiv, Sergij V. Shiyanovskii, Oleg D. Lavrentovich

**Affiliations:** 1 Liquid Crystal Institute and Chemical Physics Interdisciplinary Program, Kent State University, Kent, OH 44242 USA; 2 Department of Physics, Kent State University, Kent, OH 44242 USA

Correction to: *Nature Communications*; 10.1038/s41467-018-05101-y; published online 25 July 2018

The original version of this Article contained an error in the description of Supplementary Movie 7, which incorrectly read ‘Collision resulting in annihilation of two solitons. *U* = 45.1 V, *f* = 600 Hz, *T* = 50 °C, *d* = 8.0 μm. The original movie is taken at the frame rate of 91 fps. The playback speed is 7 fps.’ The correct version reads ‘Death of a soliton at a dust particle. *U* = 65.6 V, *f* = 800 Hz, *T* = 50 °C, *d* = 7.7 µm. The original movie is taken at the frame rate of 92 fps. The playback speed is 7 fps.’ The HTML has been updated to include a corrected version of the ‘Description of Additional Supplementary Files’ file.

