# Supplementary material for: Electrically driven three-dimensional solitary waves as director bullets in nematic liquid crystals
Source: Nat Commun. 2018 Jul 25;9:2912. doi: 10.1038/s41467-018-05101-y (PMC6060142; doi:10.1038/s41467-018-05101-y)
Supplement: Supplementary file 1 — Description of Additional Supplementary Files [file 41467_2018_5101_MOESM1_ESM.pdf]

Description of Additional Supplementary Files

File Name: Supplementary Movie 1

Description: **Director bullets as dynamic particle-like solitons.** CCN-45 cell of thickness $d = 9.3\ \mu m$, powered by AC voltage at $U = 47.4$ V $f = 250$ Hz; temperature $45\ °C$. The solitons are moving perpendicularly to the initial director which is along the horizontal direction and to the applied electric field that is normal to the field of view. Real time video rate. The width of each soliton is about 19 μm. Microscope objective 10x.

File Name: Supplementary Movie 2

Description: **Director bullets as dynamic particle-like solitons.** The same system of solitons as in movie 1, larger field of view, microscope objective 5x. Real time video rate.

File Name: Supplementary Movie 3

Description: **Nucleation of a director bullet at a dust particle.** $U = 87.4$ V, $f = 1000$ Hz, $T = 45\ °C$, $d = 8.2\ \mu m$. The original movie is taken at the frame rate of 554 fps. The playback speed is 7 fps.

File Name: Supplementary Movie 4

Description: **Nucleation and disappearance of director bullets at the edge of electrode area.** $U = 72.2$ V, $f = 1000$ Hz, $T = 50\ °C$, $d = 8.0\ \mu m$. The original movie is taken at the frame rate of 151 fps. The playback speed is 20 fps.

File Name: Supplementary Movie 5

Description: **Collision of two solitons with a small impact factor $\Delta x_{pre} < w/2$ and repulsive interaction.** 55.2 V, 800 Hz, 50 °C, 8.0 μm. The original movie is taken at the frame rate of 230 fps. The playback speed is 7 fps.

File Name: Supplementary Movie 6

Description: **Collision resulting in annihilation of two solitons.** $U = 45.1$ V, $f = 600$ Hz, $T = 50\ °C$, $d = 8.0\ \mu m$. The original movie is taken at the frame rate of 91 fps. The playback speed is 7 fps.

File Name: Supplementary Movie 7

Description: **Collision resulting in annihilation of two solitons.** $U = 45.1$ V, $f = 600$ Hz, $T = 50\ °C$, $d = 8.0\ \mu m$. The original movie is taken at the frame rate of 91 fps. The playback speed is 7 fps.

File Name: Supplementary Movie 8

Description: **Reflection of a director bullet by a dust particle.** $U = 87.4$ V, $f = 1000$ Hz, $T = 45\ °C$, $d = 8.2\ \mu m$. The original movie is taken at the frame rate of 554 fps. The playback speed is 7 fps.
